# Integrated Process Simulation of Non-Oriented Electrical Steel

**DOI:** 10.3390/ma14216659

**Published:** 2021-11-04

**Authors:** Anett Stöcker, Max Weiner, Grzegorz Korpała, Ulrich Prahl, Xuefei Wei, Johannes Lohmar, Gerhard Hirt, Martin Heller, Sandra Korte-Kerzel, Lucas Böhm, Wolfram Volk, Nora Leuning, Kay Hameyer, Rudolf Kawalla

**Affiliations:** 1 Institute of Metal Forming, TU Bergakademie Freiberg, 09596 Freiberg, Germany; max.weiner@imf.tu-freiberg.de (M.W.); grzegorz.korpala@imf.tu-freiberg.de (G.K.); ulrich.prahl@imf.tu-freiberg.de (U.P.); rudolf.kawalla@imf.tu-freiberg.de (R.K.); 2 Institute of Metal Forming, RWTH Aachen University, 52056 Aachen, Germany; xuefei.wei@ibf.rwth-aachen.de (X.W.); johannes.lohmar@ibf.rwth-aachen.de (J.L.); gerhard.hirt@ibf.rwth-aachen.de (G.H.); 3 Institute of Physical Metallurgy and Materials Physics, RWTH Aachen University, 52074 Aachen, Germany; heller@imm.rwth-aachen.de (M.H.); Korte-Kerzel@imm.rwth-aachen.de (S.K.-K.); 4 Chair of Metal Forming and Casting, TU München, 85748 Garching, Germany; lucas.boehm@utg.de (L.B.); wolfram.volk@utg.de (W.V.); 5 Institute of Electrical Machines, RWTH Aachen University, 52062 Aachen, Germany; nora.leuning@iem.rwth-aachen.de (N.L.); kay.hameyer@iem.rwth-aachen.de (K.H.)

**Keywords:** non-oriented electrical steel, simulation, grain size, texture, residual stress, magnetization, process chain

## Abstract

A tailor-made microstructure, especially regarding grain size and texture, improves the magnetic properties of non-oriented electrical steels. One way to adjust the microstructure is to control the production and processing in great detail. Simulation and modeling approaches can help to evaluate the impact of different process parameters and finally select them appropriately. We present individual model approaches for hot rolling, cold rolling, annealing and shear cutting and aim to connect the models to account for the complex interrelationships between the process steps. A layer model combined with a microstructure model describes the grain size evolution during hot rolling. The crystal plasticity finite-element method (CPFEM) predicts the cold-rolling texture. Grain size and texture evolution during annealing is captured by the level-set method and the heat treatment model GraGLeS2D+. The impact of different grain sizes across the sheet thickness on residual stress state is evaluated by the surface model. All models take heterogeneous microstructures across the sheet thickness into account. Furthermore, a relationship is established between process and material parameters and magnetic properties. The basic mathematical principles of the models are explained and demonstrated using laboratory experiments on a non-oriented electrical steel with 3.16 wt.% Si as an example.

## 1. Introduction

Non-oriented electrical steel (NO electrical steel) is widely used in electrical machines with rotating magnetic fields. Although the name indicates isotropic behavior, NO electrical steel has dominant texture components, resulting in magnetic anisotropy [1,2,3]. Furthermore, additional anisotropy can result from a residual stress distribution or an anisotropic grain size distribution. Therefore, it is necessary to use models that link material parameters and magnetic properties.

Texture and grain size are two important material parameters to consider when discussing the improvement of NO electrical steel. For example, grain boundaries interact with domain walls during magnetization and can increase magnetic losses. However, the optimal grain size is strongly dependent on frequency and applied magnetic field [3]. Concerning texture, the magnetization process sensitively depends on the crystallographic orientation. In body centered cubic (bcc) iron silicon alloys, the crystallographic 〈100〉 directions are ‘easy’ directions for magnetization and the 〈111〉 directions are ‘hard’ directions.

To approximate the final microstructure and texture, the whole process chain must be considered. For non-oriented electrical steel grades with silicon contents above 2 wt.% this is even more relevant because the material is always ferritic and does not undergo any phase transformation [4]. Accordingly, microstructure characteristics are inherited along the production and processing, influencing the final magnetic properties directly. Simulations can potentially track the microstructure development and help to determine process parameters to obtain the desired properties, potentially saving time and cost during production. In the present study, the production and processing comprises hot rolling, cold rolling, final annealing, and blanking.

The microstructure evolution during hot rolling depends on the local stress and strain as well as the strain rate, which are determined by the material flow behavior in the roll gap and the local temperature. Therefore, the material flow is affected by the rolling geometry, tribological effects and thermo-mechanical boundary conditions. The local temperature is determined by heat transfer, thermal conductivity and the conversion of plastic work to heat in the material. The microstructure evolution during hot rolling strongly depends on the aforementioned process and material properties. To predict rolling forces, the Karman equation and slab method are applicable and already used as online roll force calculation tools [5,6,7,8,9]. Furthermore, dividing the strip thickness in different layers can give local information about strain, strain rate and temperature [10]. This local information can be used to predict the microstructure at a specific height of the hot strip and enables heterogeneous microstructure prediction.

The mostly heterogeneous hot strip microstructure and texture represent the initial state for cold rolling. The evolution of grain size and texture during cold rolling is not only affected by the initial hot-rolled strip, but also by cold rolling strategies. Investigating all the influencing parameters experimentally, is time-consuming and not economic. There are many ways to simulate cold rolling processes, for example the Taylor model, advanced LAMEL (ALAMEL) model [11], Visco-Plastic-Self-Consistent (VPSC) model [12] and crystal plasticity finite-element method (CPFEM) model [13]. These models are often used to predict mechanical properties, while our goal is to simulate the cold rolling texture accurately. CPFEM is a robust method to capture the texture evolution in forming processes, even if it is relatively computationally expensive. This nonlinear numerical simulation can be realized in ABAQUS with sub-routine UMAT, or with other CPFEM toolboxes such as DAMASK [13] or WARP3D, where the constitutive models are implemented. Unlike the physics-based constitutive models that use the dislocation densities to calculate the flow stress, the phenomenological models use a critical resolved shear stress as the state variable for each slip system, which simplifies this method using a minimum number of inputs and thus making it computationally effective.

The final grain size of non-oriented electrical steel is set by the temperature-time sequence during final annealing. It has been a challenge for many years to simulate the heat treatment process with all its phenomena (recovery, recrystallization, grain growth) and implications for texture, kinetics and microstructure on a physical basis. One reason for this is that the nucleation process in the beginning of recrystallization is very complex and not conclusively understood [14]. However, it is well established that the growth is based on the grain boundary mobility and the driving forces acting on it. Generally, low angle grain boundaries have a very low mobility and high-angle grain boundaries a very high one. The grain boundary motion is thermally activated and often follows an Arrhenius function. However, it also depends on a lot of other parameters such as grain boundary character, misorientation, energy and segregation (Zener drag) whose influences are again not well understood. During recovery and recrystallization, the main driving force is the stored elastic energy (SEE), which is represented by the dislocation density. During grain growth curvature driven motion takes over resulting in minimization of grain boundary area and energy [15]. GraGLeS2D is an already established grain growth model [16]. This model is extended to the proposed heat treatment model GraGLeS2D+ to take recrystallization into account with additional driving force and nucleation. Well established numerical models to track the microstructure development during such a transformation are cellular automata [17], front-tracking [18], Monte Carlo Potts [19] or Level-set [20]. A crucial factor determining the outcome of the heat treatment simulation are the input parameters. It is important that physical parameters such as the grain boundary energy, the grain boundary mobility or the dislocation energy per unit length are well known as well as initial conditions such as the sub-grain size, the texture or the dislocation density. However, many of those parameters are not easy to access.

Following the final annealing, blanking is an essential processing step to give the final shape to the non-oriented electrical steel. Residual stress is affecting the magnetic properties of iron by hindrance to domain wall motion ([21], p. 308). Consequently, the magnetic properties of blanked electrical steel parts are deteriorated along the cut edges [22]. The knowledge of the actual residual stress state in blanked parts is of high importance to manufacturers of electromagnetic components. Although experimental measuring methods such as neutron grating interferometry, neutron diffractometry or nanoindentation are time-consuming and expensive, modeling approaches can provide an approximation of the tensile and compressive stress distribution in the blanked sheets.

The quality of NO electrical steel is determined by the magnetic properties. Therefore, all production and processing parameters are selected to improve the magnetic properties. When modeling the magnetic properties of NO electrical steel, a decoupling of the process parameters and magnetic modeling makes the magnetic models universally applicable. The requirement however is a thorough understanding of the dominant effects between the material properties and dominantly affected magnetic properties. There are semi-physical models for the description of the magnetization behavior and magnetic loss [23]. In previous work, the dominant material parameters for the magnetization and loss characteristic have been studied. Silicon content, thickness, microstructure and texture are not equally dominant for different magnetization regions or frequency ranges [24,25].

In this study, simulation tools and material models are presented for hot rolling, cold rolling, annealing, and shear cutting. Furthermore, the impact on the magnetic properties and modeling magnetic losses is demonstrated. For high accuracy, each processing step is described by an individual simulation tool with a set of process and material models. By connecting the individual tools, it is possible to describe the production and processing of non-oriented electrical steel in detail. Furthermore, predicting process parameters for defined in application properties becomes viable. As an example material, a typical composition of high silicon containing non-oriented electrical steel with 3.16 wt.% Si and 0.89 wt.% Al is used.

## 2. Material and Experimental Processing

The following sections introduce simulation tools and material models for a non-oriented electrical steel with 3.16 wt.% Si and 0.89 wt.%Al. The complete chemical composition is given in Table 1, which is a typical alloy for non-oriented electrical steel. Therefore, this material is used as an example for the applicability of the models for hot rolling, cold rolling, annealing and blanking as well as for prediction of magnetic properties. In the following sections we refer to the material by the name Fe3.2wt.%Si. Here a very brief description of the experimental procedure is given. More details are stated in the respective sections.

The material was hot rolled from a commercial 34 mm thick transfer bar to a hot strip of 1 mm thickness at the Institute for Metal Forming, TU Bergakademie Freiberg and then cold rolled to 0.5 mm and 0.25 mm at the Institute of Metal Forming, RWTH Aachen University. Final annealing was performed in a temperature range between 900 °C to 1100 °C at the Institute of Physical Metallurgy and Materials Physics, RWTH Aachen University. The annealed samples were blanked at the Chair of Metal Forming and Casting, Technical University of Munich. Magnetic measurements were conducted at the Institute of Electrical Machines, RWTH Aachen University.

## 3. Simulation of the Grain Size Distribution during Hot Rolling

**Figure 1:**
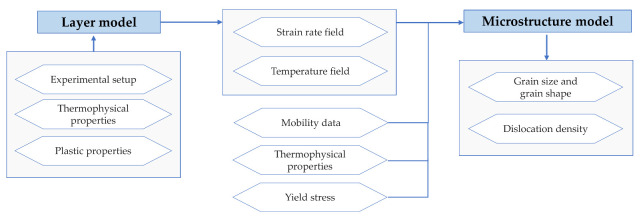
General input and output parameter for hot rolling model.

Simulating hot rolling processes focuses on rolling force and microstructure prediction. Since the microstructure evolution strongly correlates with hot rolling parameters e.g., temperature, interpass times, reduction and reduction rate per pass as well as cooling conditions, it is of great importance that the parameter values are known. To save experimental effort, simulation tools can be used to pre-evaluate specific rolling set ups. The simulation tools used here are a layer model in combination with a physical-based microstructure model. Whereby, the layer model is used for the description of the state variables temperature, flow stress and strain rate for five different positions over the strip thickness and the microstructure model uses these data to predict the local grain size distribution. Figure 1 depicts the connection of the two models.

### 3.1. Model Description

#### 3.1.1. Layer Model

The layer model is based on the classic elementary theory of plasticity, which is a well-known approach for modeling the stress distributions during rolling. This method for flat rolling is also known as strip theory or slab method, because the roll gap is divided in infinitesimal narrow strips in rolling direction on which the force balance is built. In normal direction, the strip’s height is equal to the local height of the roll gap. Each strip is deformed plane and parallelepidial. The material properties are constant in normal direction and may vary in rolling direction. Therefore, the vertical stress is also constant in normal direction and no shear deformation can be considered. The force balance at the strip element leads to the Karman differential equation [5] for the stress distribution in the roll gap. However, the strip theory assumes homogeneous material properties, stresses and strains in normal direction. To consider heterogeneous influences in normal direction, a subdivision of the strip into several distinct layers is applicable, but it is not possible to directly transfer the homogeneous approach to $N_{L}$ layers. The solution of the Karman equation depends on given strip height by the roll surface. Now, only the outer boundaries between rolling material and roll surface are known. The inner boundaries between the layers cannot be located directly. Therefore, the thickness evolution of the plastic layers must be described by an individual model. Schmidtchen [10] introduced the use of an empirical equation to distribute the total deformation to the individual layers that is based on modified equations from [26,27].

The layer model can be applied to different materials. To describe the specific material, a flow stress model depending on temperature, strain and strain rate is necessary. One applicable model is the Freiberg flow stress law as described in Equation (1) with temperature ϑ in °C, logarithmic equivalent strain φ, logarithmic strain rate $\dot{\varphi }$ and the material dependent parameters $A_{f}$ and $m_{i}$. The mean flow stress $\sigma_{f\mathrm{mean}}$ of all layers combined is calculated as the weighted arithmetic mean with height *h* of the individual layer *j* and total height of the strip $h_{\mathrm{tot}}$, Equation (2). The equivalent logarithmic strain is computed for each layer according to Equation (3) as proposed for flat deformation by Mises material law [28]; $n_{r}$ denotes the rotational frequency of the roll, $R_{r}$ the roll radius, $l_{d}$ the projected arc contact between rolls and metal, and $h_{l}$ the smallest roll gap height. For the equivalent logarithmic strain rate $\dot{\varphi }$ a mean value according to Hoff and Dahl [29] as in Equation (4) was used to simplify the iteration structure and convergence. In [8,10], the possibility of calculating local strain speed is described.

(1) $$\sigma_{f}\left(\varphi ,\dot{\varphi },\vartheta \right)=A_{f}\;\cdot \;e^{m_{1}\vartheta }\vartheta^{m_{9}}\varphi^{m_{2}}\;\cdot \;e^{m_{4}/\varphi }{\left(1+\varphi \right)}^{m_{5}\vartheta +m_{6}}\;\cdot \;e^{m_{7}\varphi }{\dot{\varphi }}^{m_{3}+m_{8}\vartheta }$$

(2) $$\sigma_{f}\;\mathrm{mean}=\sum_{j}\frac{\sigma_{f\;j}}{h_{j}}h_{\mathrm{tot}}$$

(3) $$\varphi =\frac{2}{\sqrt{3}}\log\frac{h_{0\;j}}{h_{j}}$$

(4) $${\dot{\varphi }}_{\mathrm{mean}}=\frac{2\pi \;n_{r}\;R_{r}}{l_{d}}\log\frac{h_{0}}{h_{l}}$$

The temperature evolution in each layer is computed by a simple finite differences approach that leads to a system of ordinary one-dimensional differential equations. The model is identical to the one used in [8].

Although local strain rates are not included in the flow stress model, they can be calculated afterwards from the obtained velocity field. The approach is explained in [8]. Equation (5) is derived from the strain rate tensor with the finite differences approximation of the derivatives in normal direction (ND). The velocity *v* in normal direction is approximated by Equation (6), were $\alpha_{j}$ are the angles of the respective layer boundary *j* to the horizontal line. *x* stands for the position in the roll gap in rolling direction (RD).

Several a-priori unknown parameters are included in the layer model. Consequently, a complex iteration structure is needed as described in [30].

(5) $$\dot{\varphi_{j}}=\frac{2}{\sqrt{3}}\sqrt{{\left(\frac{dv_{\mathrm{RD}\;j}}{dx}\right)}^{2}+\frac{1}{4}{\left(2\frac{v_{\mathrm{RD}\;j+1}-v_{\mathrm{RD}\;j-1}}{h_{j+1}+2h_{j}+h_{j-1}}+\frac{dv_{\mathrm{ND}\;j}}{dx}\right)}^{2}}$$

(6) $$v_{\mathrm{ND}\;j}=v_{\mathrm{RD}\;j}\;\cdot \;\tan\left(\frac{\alpha_{j}+\alpha_{j+1}}{2}\right)$$

#### 3.1.2. Microstructure Model

The applied microstructure model comprises of three interdependent models that are combined: (1) dislocation density model, (2) nucleation model, and (3) grain growth model. As a result, the volume fraction and the change in volume fraction with time are calculated for the considered grain classes. The nucleation and grain growth model are coupled, resulting in a class description of the grains for which the dislocation model is applied. Grains are calculated as unit of volume and are described by the diameter that represents the equivalent sphere of the calculated volume. A grain class has a width of 5 μm. A grain class contains grains with a grain diameter that represents the equivalent sphere with the volume of the calculated grain. Grain size and grain shape development as well as dislocation density change within each grain are considered in a representative volume element. Furthermore, each of the used attributes is evaluated statistically for every discrete time step, providing a realistic description of the microstructure [31]. As dislocation density changes permanently during hot rolling, a model is needed which considers these changes based on various effects leading to interactions of dislocations with lattice defects. An efficient model was proposed by Schacht [32], which is implemented in the microstructure model. For each grain class, the dislocation density ρ is calculated by Equation (7) with the following parameters: $c_{1}$—grain size effect parameter, $c_{2}$—dislocation density effect parameter, $c_{3}$—spontaneous annihilation effect parameter, $c_{4}$—annihilation by diffusion effect parameter, $c_{5}$—climbing speed of dislocations effect parameter, $c_{6}$—mobile dislocations effect parameter, $c_{7}$ and $c_{8}$—shearing effect parameters, $c_{9}$—activation volume effect parameter, *k*—Boltzmann constant, *R*—universal gas constant, $d_{\mathrm{sp}}$—critical distance for spontaneous annihilation, *A*—activation area, α—dislocation to dislocation interaction constant, *b*—Burgers vector, *M*—Taylor factor, $D_{s}$—self-diffusion coefficient, *G*—shear modulus, $\nu_{f}$—Debye-frequency, φ—strain, $\dot{\varphi }$—strain rate, *T*—temperature and *D(t)*—grain size.

(7) $$\frac{\partial \rho }{\partial \varphi }=A_{0}+A_{1}\sqrt{\rho }-A_{2}\rho -A_{3}\rho^{\frac{5}{2}}-A_{4}\rho^{\frac{5}{2}}\mathrm{arcsinh}\left(A_{5}\rho^{\frac{-1}{2}}\right)$$

(8) $$\begin{array}{cccc} \mathrm{with}\\ A_{0} & =\frac{M\;\cdot \;\dot{\varphi }}{b}\left(\frac{c_{1}}{D(t)}\right) & A_{1} & =c_{2}\frac{M\dot{\varphi }}{b}\\ A_{2} & =c_{3}\;\cdot \;d_{\mathrm{sp}}\frac{M\dot{\varepsilon }}{b} & A_{3} & =\frac{c_{4}c_{5}D_{s}AM\alpha b^{2}G}{kT}\\ A_{4} & =\frac{c_{4}c_{5}D_{s}AM}{c_{9}b} & A_{5} & =\frac{M\dot{\varphi }}{c_{7}b\nu_{f}}\;\cdot \;\exp\left[\frac{Q_{\mathrm{glide}}}{kT}\right]\\ Q_{\mathrm{glide}} & =\frac{c_{8}Gb^{2}}{2} \end{array}$$

Modeling grain size changes by growth and softening processes is constructed on the class-based model by Razzak [33]. Whereby, the change of grain size is expressed as a function of precipitation pinning pressure, grain boundary mobility and grain stress. Furthermore, the fraction of the grain surface is required, which is depicted by a metamodel describing the fraction as a function of the mean grain size *D* being derived from a Voronoi-mesh evaluation.

Polygonization and recrystallization are taking place during hot rolling as dynamic and static processes. The starting conditions for these processes are provided by the applied nucleation model as introduced by Korpała et al. [31]. For describing nucleation, the nucleation rate $\dot{N}$ (Equation (9)) as well as the nucleation radius $R_{N}$ (Equation (10)) are taken into account, with the following parameters: $f_{n}$—nucleation frequency, $\gamma_{\mathrm{GB}}$—surface energy of the new grain boundary, $Q_{\mathrm{diff}}$—self-diffusion activation energy, $h_{P}$—Planck’s quantum of action, $\gamma_{n}$—surface energy of the nuclei, and $P_{\mathrm{RX}}$—pressure on an individual grain boundary. The combined equations provide the control values for polygonization and the starting conditions for recrystallization.

(9) $$\dot{N}=f_{n}k\frac{T}{h_{P}}\exp\left[\frac{\frac{-\gamma_{\mathrm{GB}}}{\Delta P_{\mathrm{RX}}}+Q_{\mathrm{diff}}}{RT}\right]$$

(10) $$R_{N}=2\frac{\gamma_{n}}{\Delta P_{\mathrm{RX}}}$$

(11) $$P_{\mathrm{RX}}=\alpha G\left(T\right)b^{2}\rho$$

Additionally, conditions for the transition of sub-grain boundaries into high-angle boundaries are provided. A new grain is formed when the cumulated nucleation number reaches more than one ($\dot{N}dt>1$) and a new class is generated with volume 0.001, dislocation density 10 × 10^11^, and grain diameter 2 × $R_{N}$.

By combining the above-mentioned models as introduced by Korpała et al. [31], it is possible to describe various types of hot working flow curves under kinetic conditions with recrystallization and simultaneous grain growth. In the present study, the microstructure model is applied to calculate the grain size distribution in the hot strip for a given stress, strain, strain rate, and temperature. These necessary information at defined position in a hot strip are calculated by the layer model as described above.

### 3.2. Case Study Hot Rolling

This section illustrates the application of the models described above to simulate the microstructure evolution during hot rolling for a high silicon containing electrical steel (Table 1).

Experimentally, hot rolling was conducted within 7 passes with reductions of 55%, 50%, 48%, 46%, 28%, 20% and 16% from an initial thickness of 34 mm to a final thickness of 1 mm. As hot rolling starting temperature 1030 °C was chosen and the temperature after the last pass of hot rolling was 880 °C followed by furnace cooling with 50 K/h to room temperature. With this high finishing temperature in combination with furnace cooling, a fully recrystallized hot strip microstructure was achieved.

**Figure 2:**
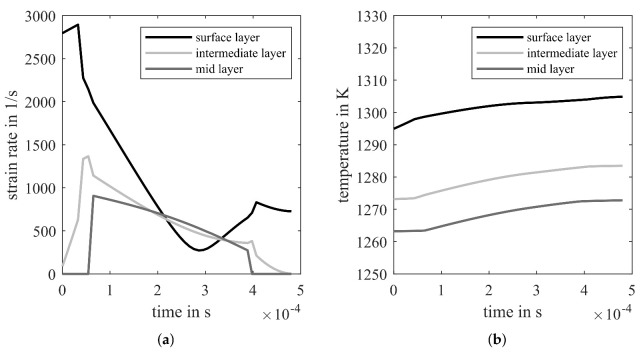
(**a**) equivalent strain rate and (**b**) temperature of surface, intermediate, and mid-layer of the hot strip in the last hot rolling pass.

To predict the grain size distribution in the hot strip, it is necessary to know the deformation and temperature development during hot rolling. The above-described layer model is used to calculate the strain rate and temperature distribution in the hot strip. Therefore, the hot strip is subdivided into five layers. Due to mirror symmetry only three layers, surface, intermediate and mid-layer, are represented. Figure 2 depicts exemplary strain rate distribution according to Equation (5) and the temperature distribution in the last pass of hot rolling. Since the microstructure model needs the time dependent strain rate and temperature distribution as input parameter, the roll gap position coordinate is converted using local material velocity to time coordinate. As a result, 0 s corresponds with the roll gap entry. The surface layer undergoes the highest strain rate which results in a stronger temperature increase caused by deformation and friction. Temperature reduction effects due to the roll contact and radiation during interpass times are neglected in the present study.

**Figure 3:**
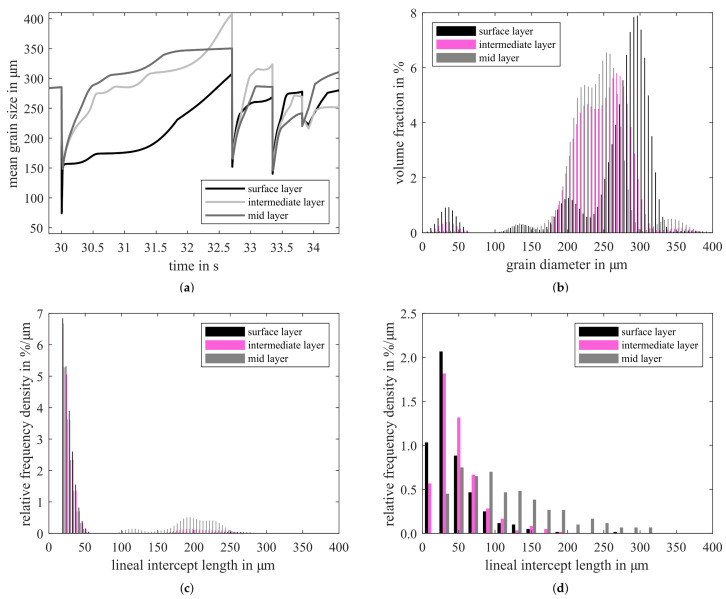
Simulated and measured grain sizes for surface, intermediate, and mid-layer of hot strip. (**a**) simulation data of the mean grain size evolution during hot rolling, (**b**) simulated grain size distribution after the last hot rolling pass, (**c**) simulated grain size distribution converted to relative frequency density and lineal intercept length, (**d**) grain size distribution of hot strip produced under laboratory conditions, grain size measured by lineal intercept method on the basis of 300 grains.

Figure 3 gives the simulated grain size as a result of the microstructure model. For the calculation the material specific data given in Table 2 were used. The evolution of the mean grain size during the last four continuous hot rolling passes is given in Figure 3a. Starting with a similar grain size across the sheet thickness, the changes in grain size across the sheet thickness become obvious. The higher strain rate at the surface leads to pronounced recrystallization effects and as a result to a smaller grain size. Besides the mean grain size, it is possible to calculate the grain size distribution, since a class-based model is used. As can be seen in Figure 3b, multimodal distributions are present after the last hot rolling pass. The different peaks are the result of the deformation steps. New grains appeared in the small grain classes because of the deformation induced nucleation and changed to higher grain classes because of grain growth at these temperatures. The grains are calculated to have a representative equivalent grain diameter and are allocated to a grain class with the respective volume fraction. To compare the simulation results to experimental data, the simulation data are converted. Based on ASTM E112-12 [34], the grain diameter is converted to a lineal intercept length and the volume fraction to a relative frequency density, see Figure 3c,d. The simulation overestimates the grains of smallest fractions. Nevertheless, the spread in grain size is represented with grain sizes of around 200 μm in the mid-layer.

The simulated grain size distribution can be used as input data for the cold rolling simulation model. Further improvements seem possible by improving the layer model to calculate local strain and strain rate across the sheet thickness. In the current approach of the layer model, the effect of shear strain is calculated based on the velocity field that results from the displacement of the layers. The local stress depends on the local strain and strain rate. Therefore, the stress would be different in a second iteration of the calculation of the deformation in the roll gap when the velocity field with shear strain is used as input data. Consequently, the local strain and strain rate would change and represent a more accurate deformation state as input data for the microstructure calculation. Besides the grain size, hot strip texture is an important material characteristic that influences the subsequent production steps. However, the here used models are up to now not able to predict hot strip texture. Additionally, the conducted literature research did not reveal any approaches of predicting the hot rolling texture for non-oriented electrical steel.

## 4. Simulation of the Microstructure Evolution during Cold Rolling

Cold rolling determines the final thickness in the production of non-oriented electrical steel. The microstructure and texture development depend on the initial hot strip and the selected cold rolling parameters. Below, an approach of predicting the cold rolling texture based on the initial hot strip texture, grain size and grain size distribution is presented.

### 4.1. Theory: Homogenization, Elastoplastic Straining, Phenomenological Constitutive Model

The approach of homogenization and the numerical solution to elastoplastic straining considered here follows the approach described by Roters et al. [13]. Therein, for the simulation of cold rolling, the isostrain homogenization is used and the plastic velocity gradient $L_{p}$ is written according to Rice [35] assuming that the deformation is only caused by dislocation slip:

(12) $$L_{p}=\sum_{\alpha =1}^{i}{\dot{\gamma }}^{\alpha }m^{\alpha }⊗n^{\alpha },$$

where ${\dot{\gamma }}^{\alpha }$ is the shear rate, *m* is the unit vector describing the slip direction and *n* is the normal to the slip plan of slip system α. The phenomenological constitutive model used is the phenopower law implemented in the CPFEM toolbox DAMASK [13], which uses critical resolved shear stresses as the state variable for each slip system. The flow law on a slip system is given by the relationship between shear rate and resolved shear stress:

(13) $${\dot{\gamma }}^{\alpha }={\dot{\gamma }}_{0}{\left|\frac{\tau^{\alpha }}{\tau_{c}^{\alpha }}\right|}^{a}\mathrm{sgn}\left(\tau^{\alpha }\right)$$

where $\tau^{\alpha }$ is the resolved shear stress, $\tau_{c}^{\alpha }$ is the critical resolved shear stress, which is also called slip resistance, and ${\dot{\gamma }}_{0}$ is the reference shear rate. The rate sensitivity of the slip system is represented by the material parameter *a*. The hardening law implemented in DAMASK describes the influence of any set of slip systems β on the hardening behavior of a fixed slip system α which empirically captures the micromechanical interaction among different slip systems:

(14) $${\dot{\tau }}_{c}^{\alpha }=\sum_{\beta =1}^{s}q_{\alpha \beta }\left[\sigma_{h}{\left(1-\frac{\tau_{c}^{\beta }}{\tau_{s}}\right)}^{w}\right]\left|{\dot{\gamma }}^{\beta }\right|$$

where $q_{\alpha \beta }$ is a measure for latent hardening, which includes entries of q = 1 for coplanar slip systems, and q = 1 or 1.4 for non-coplanar systems [36]. $\sigma_{h}$, *w* and $\tau_{s}$ are the initial hardening stress, the slip hardening exponent and the saturated flow stress, which depends on the material behavior. *s* is the total number of slip systems. In most simulations of body centered cubic (bcc) metals, two slip families with 12 slip systems each are considered that are {110}〈111〉 and {211}〈111〉.

### 4.2. Multi-Scale Simulation Scheme

**Figure 4:**
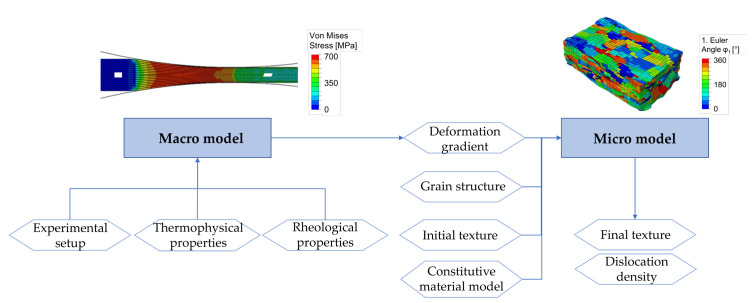
Relevant input and output data of the multi-scale simulation for texture evolution in cold rolling.

The simulation scheme consists of a macro model and a micro model. On the macroscopic scale, an isotropic hardening finite-element model in ABAQUS simulates the macroscopic strain-stress distribution that gives the deformation history in a given height layer as a boundary condition for the representative volume element (RVE) domain. Therefore, different height layers in the workpiece can be considered. The input for the macro model consists of the roll schedule, the thermo-physical properties and the rheological properties of the material. They are described in detail in the following chapters. On the microscopic scale, a crystal plasticity finite-element model is applied to every RVE domain representing a relevant portion of the workpiece that undergoes the determined deformation history extracted from the macro model and simulates the texture evolution, via a combination of ABAQUS and DAMASK. The input data for the micro model are the deformation gradient that represents the macroscopic deformation history, the grain structure, the parameters for the constitutive material model and the initial texture. Figure 4 shows the input and output data which are necessary for simulating the texture evolution during cold rolling. The dislocation density can also be an output of the micro model. However, this approach is described in detail in [37].

#### 4.2.1. Macro Model

**Figure 5:**
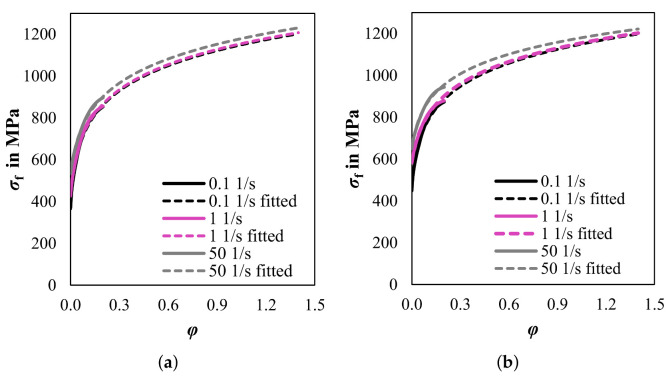
Experimental and extrapolated flow curves at room temperature determined by layer compression tests for hot strips with (**a**) a homogeneous microstructure and (**b**) a banded microstructure.

Initially, a 2D finite-element rolling model is created using ABAQUS/Standard. The simulation setup mimics an experimental cold rolling trial, i.e., rolling from an initial thickness of 1 mm to a final thickness of 0.25 mm in 6 passes as presented in [38]. The necessary elastic and thermo-physical parameters are calculated using the software JMatPro version 8.0.2 based on the chemical composition given in Table 1. For the simulation the following values at room temperature were calculated: Young’s modulus 194,108 N mm${}^{-2}$, Poisson’s ration 0.29137, conductivity 19.81 m W mm^−2^K^−1^, density 7.5764 g.cm^−3^, 459 J kg^−1^ K^−1^. As for the rheological parameters, the flow curves were obtained through layer compression tests [39] with various strain rates (0.1 s^−1^, 1 s^−1^, 50 s^−1^) on a hydraulic testing machine. To ensure that the curves are reliable, each test condition was repeated three times and only experimental data up to strains of 0.25 were used. As higher strains are required for the cold rolling simulation, all the flow curves were extrapolated to a strain of 1.4 by the method proposed in [40]. Figure 5 shows the experimental and fitted flow curves of two different hot strips. The agreement between the experimental data and the fitted data is very good.

After performing the simulations, the displacement history of nodes in the desired area of the workpiece, such as in the mid-layer or on the surface, is extracted. Based on that, the deformation gradient is calculated as input data for the micro model.

#### 4.2.2. Micro Model

On the microscopic scale, a CPFE model is generated in ABAQUS/standard using the CPFEM toolbox DAMASK [13]. Therefore, a 3D RVE with 300 grains and 17,576 hexagonal elements is created using the tessellation and meshing software NEPER [41]. The reason for using a 3D RVE is that these RVEs can better predict the cold rolling texture evolution, even in combination with a deformation gradient extracted from a 2D rolling simulation [42]. To adapt the measured texture of the hot strip to the initial texture of the RVE and to make sure that the microscopic and macroscopic material responses are in good agreement, 300 grains were identified as sufficient. The average grain size of the RVE is adjusted to match the average grain size of an experimental hot strip or a simulated one (Section 3). Furthermore, a grain size distribution can be transferred to the RVE [43]. Finally, an orientation is assigned to each of the grains based on an orientation distribution function (ODF) calculated from X-ray diffraction (XRD) measurements [44]. 300 orientations representing the macrotexture are extracted from the ODF and assigned to the 300 grains in the RVE. Thus, the RVE’s initial texture is comparable to the measured hot strip texture. The extraction method used to impose the boundary conditions onto the RVE is described in a previous study [42].

Generally, the deformation gradient obtained in the macro model is coupled with the opposite faces in the RVE to realize periodic boundary conditions (PBC):

(15) $$u_{a}-u_{b}=\left(X_{a}-X_{b}\right)\left(F-I\right)$$

Here $X_{a}$ and $X_{b}$ are the position vectors of the opposite node pairs in the RVE for the undeformed reference configuration, while $u_{a}$ and $u_{b}$ are the history of the displacement vectors for the deformed, current configuration. Auxiliary nodes are used to apply the displacement gradient *H* that is calculated from the deformation gradient *F* (H=F−I), connecting the nodes on the faces of the RVE. Additionally, the fitting of the constitutive law used in the CPFEM simulation to experimental flow curves is explained in detail in a previous study [42]. Thus, Table 3 only summarizes the data of Fe3.2wt.%Si steel with a homogeneous and a banded microstructure used in the phenopower law constitutive model together with the C${}_{11}$, C${}_{12}$ and C${}_{44}$ elastic constants for bcc metals adapted from literature [45].

### 4.3. Case Study Cold Rolling

**Figure 6:**
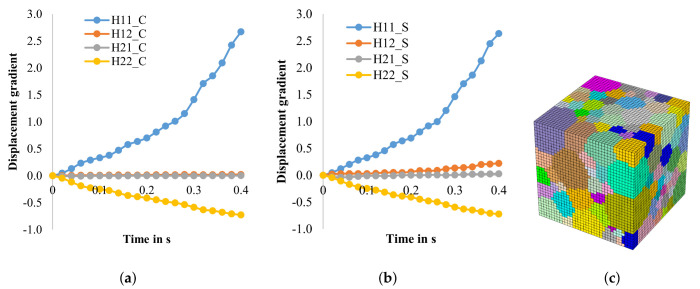
Displacement gradient in cold rolling of Fe3.2wt.%Si with a homogenous microstructure, (**a**) in the mid-layer and (**b**) near the surface. (**c**) Corresponding RVE with 300 grains used in both cases. 11—displacement gradient in normal direction, 22—displacement gradient in rolling direction, 12 and 21—displacement gradient indicating shear, C—mid-layer, S—near surface layer.

In a previous study [46], it was found that the development of texture near the surface and in the mid-layer is different during cold rolling. To analyze this further, the aforementioned CPFE model was used to predict the position dependent texture evolution for a fully recrystallized hot strip that comes with the initial grain size and flow curve. The deformation gradient for both height layers (surface and mid-layer) is obtained from the macro model and transformed into a displacement gradient that is then imposed on the auxiliary nodes of the micro model as a boundary condition. In the macro model, cold rolling with six rolling passed to 0.25 mm with a total reduction of 75% is implemented. The displacement gradients applied are shown in Figure 6. Although they are broadly similar, a clear difference is visible in the H12 component representing shear. There is a clear shear influence visible near the surface, while no shear is visible in the mid-layer.

**Figure 7:**
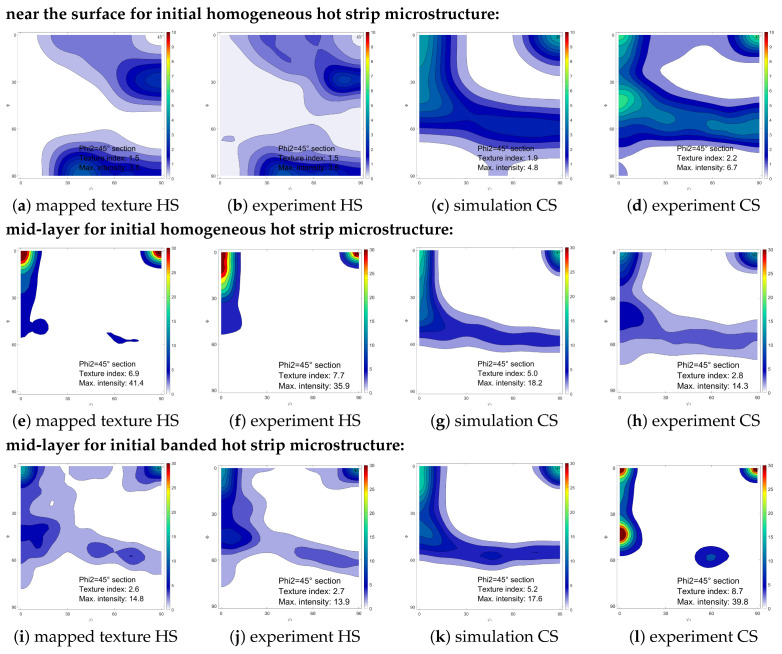
Comparison of experimental and simulation results for the texture evolution in Fe3.2wt.%Si steel. (**a**–**d**) Measured and predicted texture evolution near the surface of a homogeneous hot strip microstructure, (**e**–**h**) Measured and predicted texture evolution in the mid-layer of a homogeneous hot strip microstructure, (**i**–**l**) Measured and predicted texture evolution in the mid-layer of hot strip with a banded microstructure. HS—hot strip, CS—cold strip.

Figure 7 gives the simulation and experimental texture results, in the mid and surface layer. Compared to the experimental texture of the hot strip, the mapped initial texture is in good agreement for both layers. Furthermore, after cold rolling to the final thickness of 0.25 mm, the simulated cold-rolled texture reflects the experimental texture evolution well. In the mid-layer, the measured texture reveals a transformation of rotated cube texture into α and γ-fiber, while the texture intensity is weakened. For the near surface layer, the Goss texture and close by orientations change during cold rolling to α-fiber, γ-fiber and rotated cube. For both areas, the simulation reveals the same texture changes. However, the quantitative intensity increase or decrease is not fully captured.

**Figure 8:**
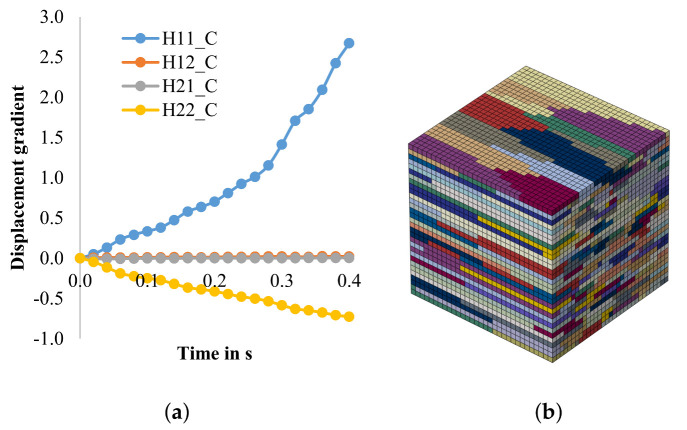
(**a**) displacement gradient during rolling of Fe3.2wt.%Si with a banded microstructure in the mid-layer, (**b**) corresponding RVE with 300 grains.

In another previous study [47], the texture development during cold rolling of hot strips with different textures were investigated. Therefore, it is important for the cold rolling model to have the ability to simulate the texture evolution based on different initial hot strip states. In the following, a Fe3.2wt.%Si hot strip with a banded structure is used as initial state. Based on the flow curves (Figure 5), the parameters for phenomenological model are fitted, see Table 3. The RVE with band structure was created in NEPER, and the displacement gradient in the mid-layer was calculated (Figure 8).

The experimental hot strip texture is mapped to an RVE with a banded grain structure with a high degree of conformity, see Figure 7i,j. In the simulated result, the cold strip texture is not as greatly intensified as in the experiment, but the tendency of α-fiber and ND-Cube strengthening is captured.

As exemplary shown above, it is possible to predict the texture evolution in different layers of the sheet. Therefore, different initial textures and grain shapes can be considered. By that, it is possible to take a wide variety of initial hot strip structures and textures into account. Furthermore, based on the simulations, an estimation for suitable hot strip states for a tailor-made material can be made.

## 5. Simulation of the Microstructure Evolution during Final Annealing

### 5.1. Model Description

Given the fact that the magnetic properties of non-oriented electrical steel strongly depend on grain size, texture, and internal defects like dislocations, annealing after cold rolling is a crucial production step. To model the annealing process, a level set method is implemented [16,48]. The foundation of this approach is in signed distance functions that are assigned to each interface *S*,

(16) S={x∈Ω∣Φ(0,x)=0}

where position *x* is part of a defined computational domain Ω and Φ(0,x)=0 is the zero level-set of this real-valued function. In order to keep the algorithm efficient, the level-set function needs to fulfill the following criterion,

(17) ∣Φ(t,x)∣=1

where *t* is an implicit time value. The following flow chart describes the main steps of the model.

**Figure 9:**
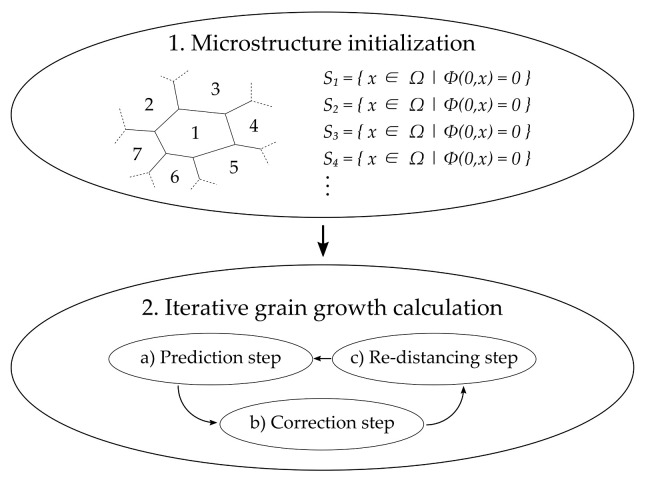
Flow chart showing the main steps of the level-set method.

First, the microstructure needs to be initialized. In this context, every grain receives its own zero level-set signed distance function Equtaion (16) representing the entire grain and grain boundary during simulation. Next, grain growth is calculated in an iterative manner. Therefore, three steps (Figure 9a–c) are repeated until an abort criterion is fulfilled. Initially (Prediction step), the movement of each individual grain boundary is calculated based on the driving forces acting on it, before each movement is corrected based on individual anisotropy effects, in our case grain boundary energies, grain boundary mobilities and triple junction mobilities. Due to this individual consideration of grain boundary movements, there are overlaps and voids in the microstructure after this initial step that are corrected in the Correction step. In order to start with the Prediction step again, the level-set functions of each grain needs to be reinitialized (Re-distancing step) to their zero level-set signed distance functions Equtaion (16). One big advantage of this procedure is that it allows parallel computation. For a detailed description of the approach, the reader is referred to [16,48]. In the following section, the focus is on the actual use of the model as well as on a case study. The initial microstructure can either be directly taken from the cold rolling model described above Section (Section 4), an EBSD measurement where values for dislocation densities and subgrains need to be added or it can be artificially created through an optimized microstructure generator [49], where parameters like subgrain size, subgrain orientation, texture, dislocation density and grain shape can be assigned manually. In the following section, the latter is used.

### 5.2. Determination of the Input Parameters

To account for the inhomogeneous grain interior after rolling, the microstructure generator not only generates elongated grains (parent grains) but also sub-grains within these grains that can scatter in orientation and dislocation density to a certain extent (SubGrainScatter). The following tables contain the most important input parameters for the microstructure generator [49] and the GraGLeS2D+ [50] model used in the following case study. The results from Wei et al. [38] serve as an experimental basis.

In the present case study, three heat treatment temperatures were simulated (900 °C, 1000 °C, 1100 °C). A heating or cooling ramp was not considered. Macroscopically, the initial cold rolling microstructure and texture of all cases were the same. For the dislocation density of the nuclei (NucleiSEE) and the deformed matrix (QuantitativeSEE), typical values of an annealed and modest to heavily deformed state were taken respectively based on literature [14]. Additionally, as has been found in experiments [44], different texture components of the deformed matrix were assigned different dislocation densities starting from the aforementioned density (QualitativeSEE). Lastly, the dislocation densities of the parent grains’ subgrains were sampled from normal distributions with a variance of 0.1 around the respective parent grain value. The maximum sub-grain scatter was estimated to be 3°, which should be related to the sub-grain size (physical domain size/number of sub-grains) and the degree of deformation. Both could be estimated from an EBSD map. A problem of this approach is that, in reality, grains often show an increasing orientation gradient from one side of the grain to the other that can be above 3°. This can currently not be considered. The initial mid-layer texture was provided through an XRD measurement in the RD-TD plane of the cold-rolled sheet (see [38,44]). After rolling, the grains are usually elongated, often with a height-to-length ratio way below 0.01; however, the microstructure generator was only able to generate a sensible microstructure up to a value of 0.04. Physical material properties and their respective theoretical models, such as the anisotropic (misorientation dependent—θ) high-angle grain boundary energy γ depicted by the Read and Shockley equation Equation (18) [51] (HAGB_Energy) or the dislocation energy per meter (DislocEnPerM), were directly taken from literature.

(18) $$\gamma =\left\{\begin{array}{cc} \frac{\theta }{15}\left[1-\ln\frac{\theta }{15}\right] & \mathrm{for}\;\theta \leq 15{}^{\circ}\\ 1 & \mathrm{for}\;\theta >15{}^{\circ} \end{array}\right.$$

Since the high-angle grain boundary mobility (HAGB_mobility $\mu_{\mathrm{GB}}$) is one of the most important parameters, especially for the time and temperature dependent interpretation of the simulation results, it was first taken from literature [52,53], but also reviewed experimentally [44]. Since this value determines the growth behavior and is strongly temperature dependent, it is different in all three cases (Table 4 and Table 5). Moreover, this value also depends on the grain boundary character. Small angle grain boundaries have a very low mobility and high-angle grain boundaries a high one. To account for this, a mobility model is applied (see Equation (19)) where the mobility is low for angles below 8° and increases exponentially before reaching a plateau above 15° at the selected value (QuantitativeSEE).

(19) $$\mu_{\mathrm{GB}}=1.0-\left(0.99\;\cdot \;\exp\left[-5\;\cdot \;{\left(\frac{\theta }{15{}^{\circ}}\right)}^{9}\right]\right)$$

This model can and should be adapted to new findings. For example, it is not clear whether selected boundaries (sigma boundary, specific axis etc.) have specific mobilities. Moreover, the influence of temperature and alloying elements is not quantified yet and it is not clear whether the mobility is lower at very high misorientation angles again [56,57].

The triple junction drag (TripleJunctionDrag) is currently dimensionless and rather empirical. However, literature [58] showed that it has an influence which is already implemented into the code here in a basic fashion and can be further improved in the future. The number of points per grain (NrOfPPG) is a mathematical value where the optimum between accuracy and computing time needs to be found, which has been done in [55].

Overall, to generate a realistic cold rolling electrical steel sheet microstructure ready for heat treatment simulation, a macrotexture and a rough idea of the microstructure (grain shape, dislocation density distribution, sub-grain size/scatter, nuclei density) is needed. The biggest problem are the nuclei. As mentioned above, the nucleation process is not well understood. Thus, there are no straightforward correlations for the nuclei density, position or orientation. It is just vaguely assumed that nuclei are formed during deformation and recovery and are already preexisting before recrystallization [14]. Currently, some sub-grains are randomly replaced by nuclei with a specific probability in the microstructure generator. A low dislocation density and a by XRD measured texture is assigned to those nuclei. The texture is taken from an experimental heat treatment where recrystallization has just finished. Since the nucleation process is more or less left out and the incubation time before recrystallization is extremely low (< 5 s) at an appropriate temperature (<900 °C) for the examined material, leaving out the recovery process during simulation seems justifiable here. As soon as the understanding of the nucleation process is improved, this should be implemented into the model. It is also well known that the nucleation rate depends on the temperature [14]. The higher the temperature the higher the nucleation rate. However, finer adjustments taking the relationship between nucleation rate and growth rate into account or an orientation-dependent nucleation rate can currently not be made, although this would be important for a physical model, because again the detailed knowledge is missing. In our case study, the nucleation density is the only parameter we can use to account for the complex topic of nucleation. Regarding the nuclei, it has also been found during our studies that they need to have a minimum size (> 2 μm) within the model to be able to grow.

### 5.3. Case Study Annealing

In the following section, simulation results (kinetic, microstructure and texture development) will be validated by individual experimental results (see [38]). Therefore, the experimental annealing process of a 0.25 mm cold strip annealed at 900 °C to 1100 °C will be compared to simulation results in depth.

**Figure 10:**
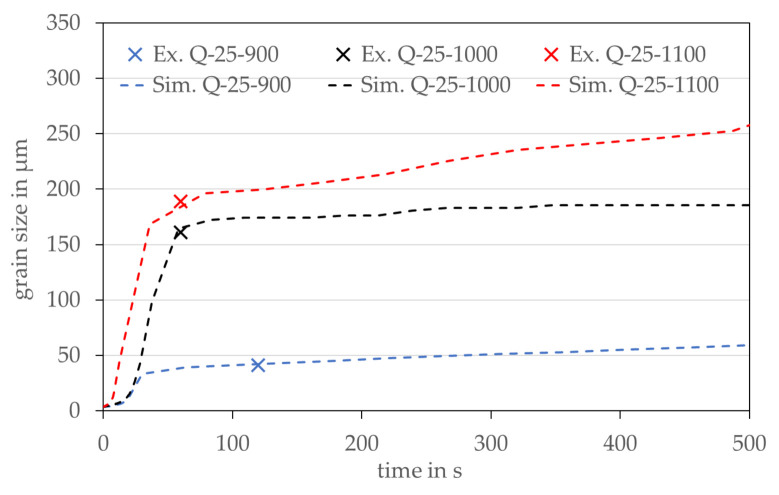
Simulated recrystallization and grain growth kinetics (dashed lines) based on grain size (y-axis) and time (x-axis) compared to individual experimental results (crosses). Data for experimental results are taken from [38].

As can be seen in Figure 10, there is always a unique point at which the slopes of the grain size—time curves change. This point can be correlated with the time where the randomly distributed and fast growing nuclei touch each other. In the following, the first section will be called recrystallization (RX) section and the second one grain growth (GG) section. Within the RX section, mainly the randomly distributed nuclei grow very rapidly after a short incubation time due to the here dominating SEE driving force. As soon as the nuclei with their low SEE touch each other and all grains have more or less the same SEE this driving force vanishes. From this point, the always present driving force to minimize the grain boundary energy through minimizing the grain boundary curvature becomes the dominant driving force, which is directly correlated with grain growth. It is recognizable that the grain size results of all three annealing experiments could be reproduced perfectly. However, the nuclei density was determined empirically to match the experimental data point. In the future, it would be desirable to experimentally confirm entire curves, rather than individual points as shown in Figure 10, so that with greater confidence in the actual position of the changes in slope of the grain size-time relationship, the best annealing time can be identified with respect to the application dependent grain size. Current developments in the use of in-situ, and therefore much more continuous, characterization of grain size and texture over time [44] suggest that this should indeed be possible in the near future.

**Figure 11:**
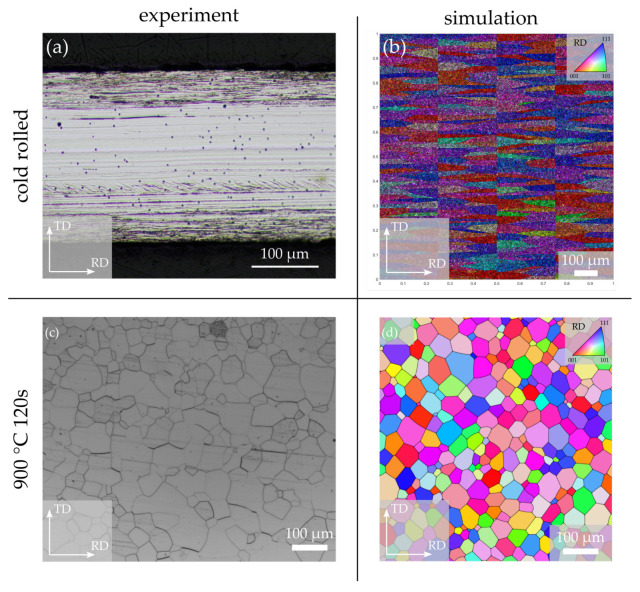
Light microscopy images of (**a**) the cold rolled and (**b**) the sheet annealed at 900 °C in comparison to the simulated microstructures (**c**) using the microstructure generator to produce the equivalent to the cold-rolled state and (**d**) the resulting microstructure after simulation of RX and GG.

**Figure 12:**
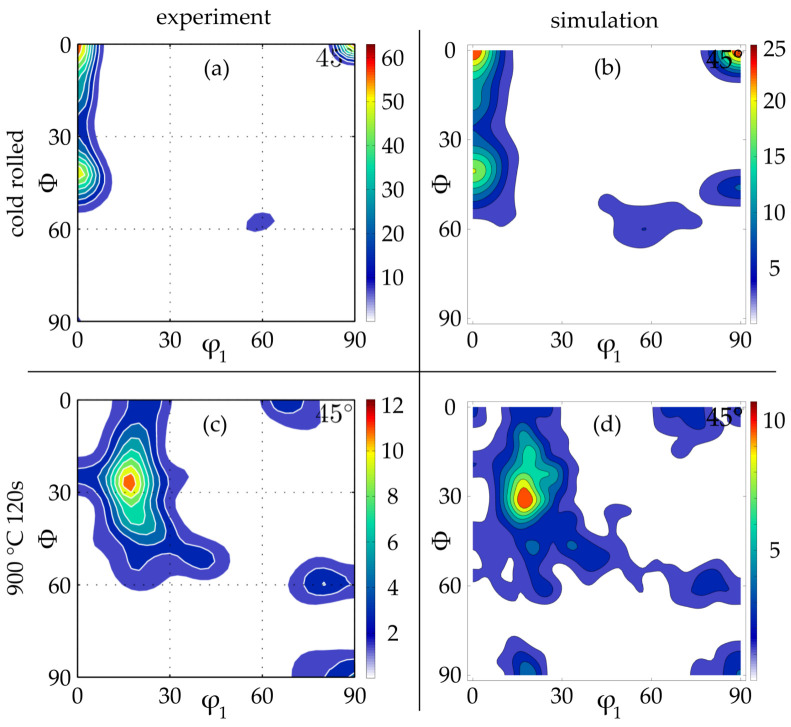
ODF $\varphi_{2}$ = 45° section of the experimental (**a**) and simulation (**b**) cold rolling texture as well as the resulting experimental (**c**) and simulated (**d**) texture after annealing for 120 s at 900 °C.

Figure 11 compares the microstructure results before and after annealing and Figure 12 shows the macrotexture results. The RD–ND plane shown in Figure 11a is especially interesting in electrical steel because it often directly shows the typical differences across the sheet thickness [44]. In the layer of the sheet that is close to the surface additional shear deformation through friction between sheet and roll adds additional deformation and finally results in a higher dislocation density, different texture components and more deformation structures such as shear and deformation bands [59]. These different conditions before annealing can also result in different microstructures, textures, and magnetic properties along the sheet thickness after annealing. In the presented case study, we investigate the RD–TD mid-layer because layer differences cannot be generated with the microstructure generator at the moment. Beside the additional deformation and deformation structures close to the surface (Figure 11a—darker areas) no vertical grain boundaries can be seen in the mid-layer, which means that the height-to-length ratio of the grains is extremely low. Unfortunately, the minimum value within the microstructure generator, which still generates sensible microstructures, is 0.04. The resulting microstructure with its orientation gradients in the parent grain/sub-grain structure can be seen in Figure 11b. Obviously, the microstructure generator should be further improved to consider more realistic microstructures (orientation gradients, height/length ratios, deformation structures) and nucleation (orientation relations, position, number) in the future. After annealing, the experimental (Figure 11c) and simulated (Figure 11d) microstructures look quite similar. The grains are equiaxed and qualitatively of the same size.

With regard to the macrotexture, the cold rolling, Figure 12a,b, as well as the annealing, Figure 12c,d, $\varphi_{2}$ = 45° ODF sections fit very good between simulation (Figure 12b,d) and experiment (Figure 12a,c). At least for the cold-rolled texture this is no surprise since the microstructure generator uses Figure 12a as an input; however, this comparison still proves that everything went right during the generation. Next, the good fit after annealing was also expected since the nuclei, which consume the deformed microstructure within the RX section, were given exactly the texture from Figure 12c as an input. Nevertheless, the good fit at exactly this time step underlines the relevance of Figure 10, since some texture components vanish during further growth, theoretically resulting in a worse match.

Overall, we provide here a comprehensive parallelized simulation model framework with many initial approaches to simulate the heat treatment process taking recrystallization and grain growth into account. The two most important driving forces for recrystallization (SEE) and grain growth (curvature driven) are considered and a smooth transition between the two phenomena is achieved. Moreover, the most important physical properties of grain boundaries are taken into account (energy, mobility). However, further work needs to be done. Nucleation is not yet well understood and thus could not be implemented perfectly into the model. Furthermore, the mobility model as well as the microstructure could be depicted more realistically and the triple junction drag is just empirically right now.

## 6. Simulation of Residual Stress after Shear Cutting

**Figure 13:**
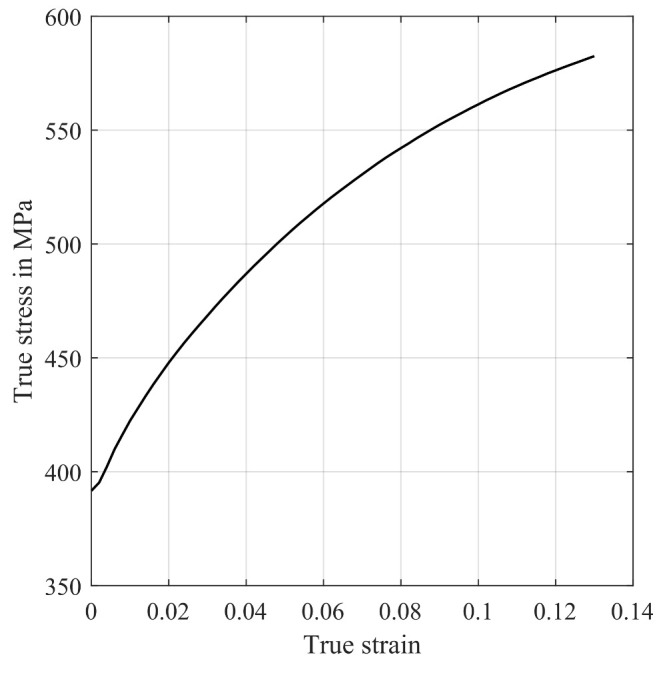
Experimental flow curve for the examined electrical steel.

Although cold rolling determines the final thickness and annealing the final grain size and texture, shear cutting determines the final shape of non-oriented electrical steel. Therefore, the magnetic properties are deteriorated by residual stresses caused by cutting along the cut edges. The simulation of evolving residual stress is an approximative alternative to time-consuming measuring approaches. In comparison to other steel grades, electrical steel typically has a small sheet thicknesses and large grains. Considering the fact that grains on the surface of the sheet feature less resistance against deformation than inner grains, the assumption of a homogeneous material is not sufficient. Other authors [60,61,62] developed the idea of the so-called surface model for the simulation of materials with an unneglectably high relation of surface grains to inner grains. The total number of grains *N* in a sample is divided into surface grains $N_{\mathrm{sg}}$ and inner grains $N_{\mathrm{ig}}$ which are then assigned different material properties [61]. In this section, the surface model is applied to simulate the shear cutting process of electrical steel and the resulting residual stress state in the blanked material. The mechanical properties (Table 6) of the 0.5 mm thick material were measured by a standardized uniaxial tensile test. Figure 13 shows the corresponding flow curve.

### 6.1. Description of the Surface Model

According to the surface model, the flow stress σ of a material is composed of the flow stress of its surface grains $\sigma_{\mathrm{sg}}$ and inner grains $\sigma_{\mathrm{ig}}$, which is described by Equation (20). The flow stress of surface grains resembles to that of single crystals and can thus be approximated by Schmid’s law, which leads to Equation (21) with the orientation factor m ≤ 2 and $\tau_{R}\left(\varepsilon \right)$ denoting the critical resolved shear stress. On the other hand, the flow stress of inner grains is similar to the flow stress of a polycrystal, therefore the Hall-Petch equation applies, where *M* is the Taylor factor related to the slips systems in Equation (22). The relation between the number of surface grains to the number of inner grains is described by the scale factor η in Equation (23) [61]. Combining Equations (20)–(23), Equation (24) follows for the flow stress of the material according to the surface model.

(20) $$\begin{array}{ccc} \sigma & = & \frac{N_{\mathrm{sg}}\;\cdot \;\sigma_{\mathrm{sg}}+N_{\mathrm{ig}}\;\cdot \;\sigma_{\mathrm{ig}}}{N} \end{array}$$

(21) $$\begin{array}{ccc} \sigma_{\mathrm{sg}}\left(\varepsilon \right) & = & m\;\cdot \;\tau_{R}\left(\varepsilon \right) \end{array}$$

(22) $$\begin{array}{ccc} \sigma_{\mathrm{ig}}\left(\varepsilon \right) & = & M\;\cdot \;\tau_{R}\left(\varepsilon \right)+\frac{\kappa (\varepsilon )}{\sqrt{d}} \end{array}$$

(23) $$\begin{array}{ccc} \eta & = & \frac{N_{\mathrm{sg}}}{N} \end{array}$$

(24) $$\begin{array}{ccc} \sigma (\varepsilon ) & = & \left(M+\eta \;\cdot \;(m-M)\right)\;\cdot \;\tau_{R}\left(\varepsilon \right)+\left(1-\eta \right)\;\cdot \;\frac{\kappa (\varepsilon )}{\sqrt{d}} \end{array}$$

The examined material has a mean grain size *d* of 117 μm and a sheet thickness of 0.5 mm, which results in $\eta =\frac{2\;\cdot \;117}{500}=0.468$. Following the assumption that m = 2 and M = 3.06 [61], the flow stress of the examined material is given by Equation (25).

(25) $$\sigma \left(\varepsilon \right)=2.56392\;\cdot \;\tau_{R}\left(\varepsilon \right)+0.532\;\cdot \;\frac{\kappa (\varepsilon )}{\sqrt{d}}$$

The critical resolved shear stress $\tau_{R}\left(\varepsilon \right)$ and the locally intensified stress needed to propagate yield across grain boundaries divided by the square root of the grain size $\frac{\kappa (\varepsilon )}{\sqrt{d}}$ remain undetermined variables, which will be approximated in the following section.

### 6.2. Separation of the Flow Curve

**Figure 14:**
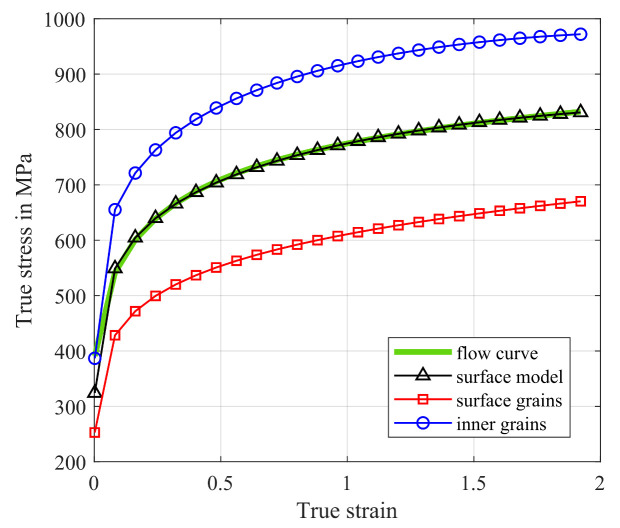
Separation of the extrapolated flow curve in the flow stress of surface grains and inner grains.

First, the experimental flow curve is extrapolated to higher strains that are present during the shear cutting experiment with a combination of 50% Hockett-Sherby and 50% Swift hardening model. Then, it is assumed that the undetermined variables $\tau_{R}\left(\varepsilon \right)$ and $\frac{\kappa (\varepsilon )}{\sqrt{d}}$ from Equation (25) can be approximated by quadratic approaches as shown in Equation (26). The combination of Equation (25) and (26) results in Equation (27), which represents a set of equations for the flow stress of the material at increasing strain levels up to a maximum strain of the extrapolated flow curve of ε = 2. Equation (27) is solved for all strains with the Least Squares Method in Matlab and the strain dependent values for $\tau_{R}\left(\varepsilon \right)$ and $\frac{\kappa (\varepsilon )}{\sqrt{d}}$ are obtained from Equations (28) and (29). Figure 14 shows the approximation of the extrapolated flow curve with the surface model and the separation of surface flow stress and bulk flow stress according to Equations (21) and (22).

(26) $$\begin{array}{ccc} \tau_{R}\left(\varepsilon \right)\;\mathrm{or}\;\frac{\kappa (\varepsilon )}{\sqrt{d}} & = & a\;\cdot \;\varepsilon^{b} \end{array}$$

(27) $$\begin{array}{ccc} \sigma (\varepsilon ) & = & 2.56392\;\cdot \;a\;\cdot \;\varepsilon^{b}+0.532\;\cdot \;c\;\cdot \;\varepsilon^{d} \end{array}$$

(28) $$\begin{array}{ccc} \tau_{R}\left(\varepsilon \right) & = & 305.4587\;\cdot \;\varepsilon^{0.1420} \end{array}$$

(29) $$\begin{array}{ccc} \frac{\kappa (\varepsilon )}{\sqrt{d}} & = & -15.5430\;\cdot \;\varepsilon^{1}.8914 \end{array}$$

### 6.3. FEA Modeling of the Shear Cutting Process

**Figure 15:**
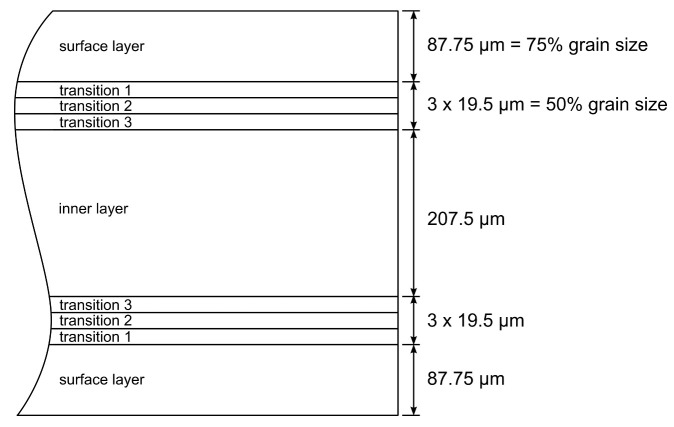
Sectioning of the electrical steel sheet for the FEA of the shear cutting process.

To simulate the shear cutting process, a 2D simulation in Abaqus FEA is realized by following the description by Weiss et al. [22]. It is based on the experimental shear cutting tool which was first presented by Weiss et al. [63]. The sheet is meshed with elements of the type CPE4R, which is a 4-node bilinear plane strain quadrilateral element with reduced integration and hourglass control. The length of the element edges is 0.005 mm in the shear zone and 0.05 mm in the remaining part of the sheet. The arbitrary Lagrange-Eulerian method is used for elements in the shear zone every third time increment. Punch, blank holder and die are modeled as rigid bodies. Friction is modeled with a friction coefficient of 0.16 between tool parts and sheet. The cutting-edge radius of the punch and die is 10 μm, the cutting clearance equals to 50 μm which is 10% of the sheet thickness. New to the finite-element analysis (FEA) model is that the sheet no longer consists of a homogeneous material but is divided into a total of nine sections with a varying stress-strain behavior. The surface layer of the sheet features the stress-strain data of surface grains, the inner layer of the sheet features the stress-strain data of inner grains and three different transition layers feature mixed stress-strain data. The transition layers were chosen regarding model a smooth transition between surface grains and inner grains. Figure 15 shows the division of the electrical steel sheet in these nine sections. In the different sections of the sheet, the composition of the flow curve is as follows: surface layer—flow curve of surface grains; transition 1—flow curve of 2/3 surface grains and 1/3 inner grains; transition 2—flow curve of 1/2 surface grains and 1/2 inner grains; transition 3—flow curve of 1/3 surface grains and 2/3 inner grains; inner layer—flow curve of inner grains.

**Figure 16:**
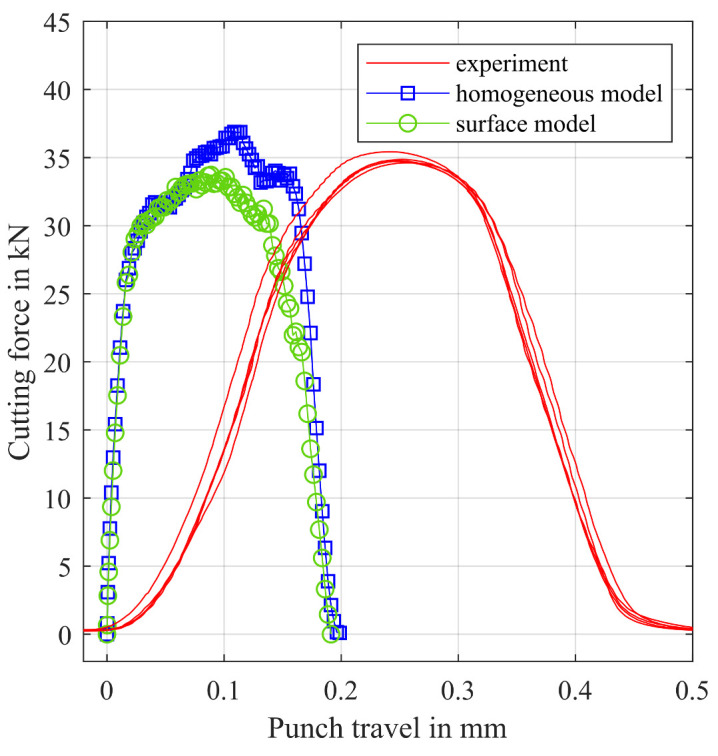
Cutting force over punch travel of the shear cutting experiment as well as of the FEA with surface and homogeneous model.

Electrical steel samples of the evaluated material are shear cut with an experimental shear cutting tool on the mechanical Bruderer press BSTA510-125. The cutting force-punch travel curve was measured during the punching operation. These data were used to calibrate the simulation model by multiplying the flow curves of the different sections with a best fit scaling factor of 1.4 and by adjusting the damage criterion. Figure 16 shows the simulated and smoothed punch force over punch travel for the surface model and the homogeneous model as well as the smoothed experimental punch force-punch travel curves. The slope of the simulated curves is much higher since the stiffness of the press machine was neglected in the FEA. Apart from that, the surface model results in a typically shaped cutting force-punch travel curve of the shear cutting process.

### 6.4. Evaluation of Residual Stress State

**Figure 17:**
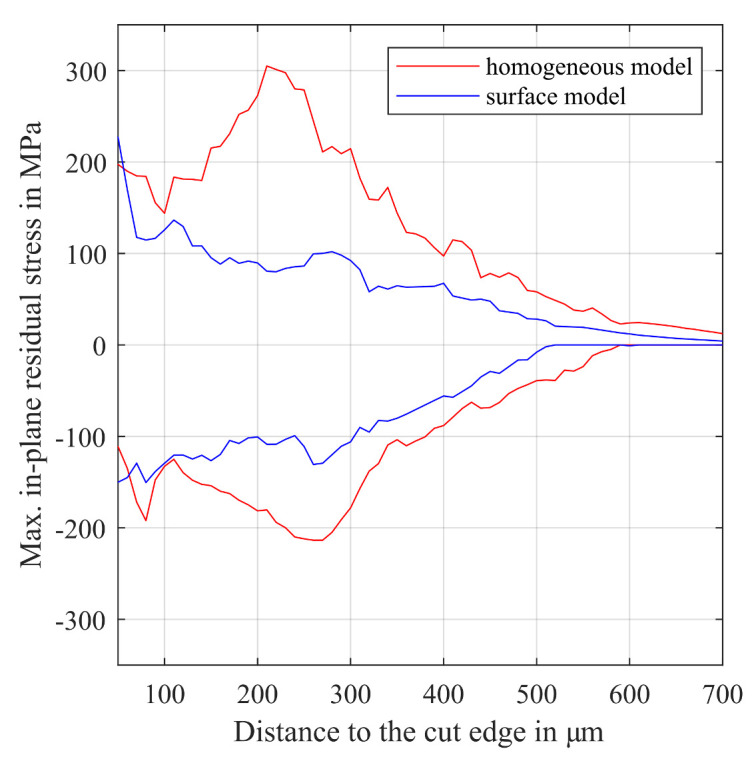
Residual tensile and compressive stress in the blanked electrical steel sheets according to the surface and homogeneous model.

The evaluation of residual stress state follows the description in [22]. The maximum in plane absolute principal tension and compressive stress for increasing distances to the cut edge are evaluated. Figure 17 shows the residual stress state for the homogeneous model and the surface model in the zone affected by shear cutting. The calculated residual stress state for the surface model shows a plausible distribution with decreasing stress values for an increasing distance from the cut edge. The penetration depth of residual stress into the material is in good accordance with NGI experiments on blanked electrical steel [64]. In comparison to the homogeneous material model, the application of the surface model results in a peak stress directly at the cut edge and generally lower stress levels.

## 7. Prediction and Correlation of Magnetic Properties

In the preceding models, the effect of hot rolling, cold rolling and annealing on the texture and microstructure evolution, as well as the effect of shear cutting on residual stress state has been described. When modeling the magnetic properties of NO electrical steel, a decoupling of the process parameters and magnetic modeling by directly linking magnetic properties to material parameters makes the magnetic models universally applicable. Not only can the magnetic properties be modeled when the production route is known, and texture and microstructure are modeled, but the magnetic models can also be parameterized when the relevant material properties are obtained by measurements from fully finished sheet material.

In the following section, two models are described that allow the implementation to finite-element simulations of electrical machines. With these semi-physical models, which link material parameters to magnetic properties, the impact of different materials during the application of electrical steel in electrical machines can be evaluated. The first model describes the magnetization anisotropy as a function of crystallographic texture and silicon content. The second model includes the effect of microstructure, thickness and alloying content on magnetic loss.

### 7.1. Texture Model to Describe the Magnetic Anisotropy of Non-Oriented Electrical Steel

Although classified as non-oriented, as shown in the previous sections and various scientific literature, electrical steel has dominant texture components and a resulting magnetic anisotropy [1,2,3]. This anisotropy can further be affected by the residual stress distribution or an anisotropic grain size, i.e., elongated grains. The semi-physical anisotropy model presented in [65,66] allows the description of magnetization curves in every direction of the RD-TD-sheet plane. For the parametrization, three magnetic measurements in rolling direction (RD), transversal direction (TD) and 55° relative to RD as well as the ODF of the material are required. The magnetic field strength *H* for any spatial direction θ in the sheet plane relative to RD and magnetic polarization *J* can be calculated at a constant frequency as follows [66]:

(30) $$\begin{array}{c} H_{\mathrm{model}}\left(J,\theta \right)=\omega H_{\mathrm{texture}}+\left(1-\omega \right)H_{\mathrm{eliptical}}\\ \mathrm{with}\;\omega \;\mathrm{so}\;\mathrm{that}\;|H_{55,\mathrm{meas}.}-H_{55,\mathrm{model}}|\to min.\;\mathrm{for}\;\mathrm{every}\;J \end{array}$$

**Figure 18:**
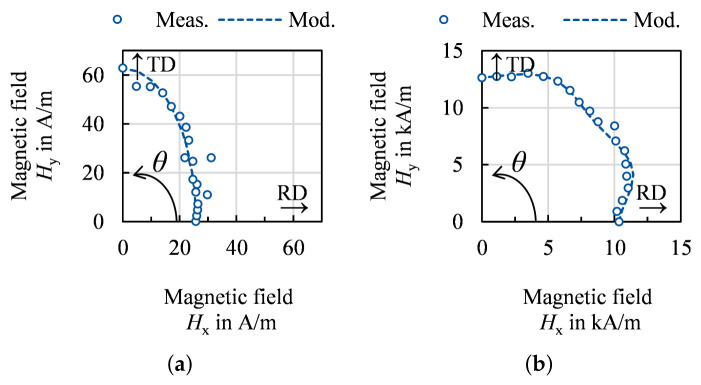
Comparison of continuously modeled and in 5°-steps measured magnetic field in the RD-TD plane at two different polarizations for a commercial M235-35A steel at 50 Hz, (**a**) at 0.3 T, (**b**) at 1.8 T.

The model couples an elliptic model component with a texture model component through a weighting factor ω. The elliptical model component only needs to be parametrized with magnetic measurements in RD and TD. It describes the polarization in the $H_{\mathrm{RD}}-H_{\mathrm{TD}}$ plane as an ellipse. This elliptical behavior occurs mainly for low to medium polarizations as seen in Figure 18a [65,66]. According to the domain theory, this magnetization range is linked to the domain wall movement [24]. The movement of domain walls is generally sensitive to residual stress, grain size and surface defects [24,25]. As grains are almost spherical for annealed electrical steels, the residual stress from rolling, although being small is likely the cause for the elliptical behavior with a small tensile stress in RD and a small compressive stress inTD. For the texture model component, the course of the so-called A-parameter *A* [2] needs to be evaluated. This parameter is solely calculated from the ODF and can continuously be described by a polynomic function [66]. Scaled to the RD and TD measurements, the course describes the texture related required magnetic field strength in every spatial direction.

(31) $$H_{\mathrm{texture}}\left(J,\theta \right)=H_{\mathrm{RD}}\left(J\right)+A_{\theta }\left(\frac{\Delta H_{\mathrm{scale}}\left(J\right)}{\Delta A_{\mathrm{scale}}}\right)-A_{\mathrm{RD}}\left(\frac{\Delta H_{\mathrm{scale}}\left(J\right)}{\Delta A_{\mathrm{scale}}}\right)\;$$

with ΔH and ΔA being the absolute amount of the difference of *H* and *A* in TD in relation to RD. The weighting factor ω links the texture component to the elliptical model, whereby ω increases with *J* because in the range of medium and high polarization the texture effect becomes dominant. In this polarization range, the magnetization process occurs as a result of domain rotation. Magnetic moments are initially aligned to easy crystallographic directions but now align with the external magnetic field. More magnetic field is required if the easy crystallographic axes have a large misorientation to the magnetization direction [2,24]. Thus, the effect of texture becomes dominant. In Figure 18b at 1.8 T the dominant effect of texture can be seen.

On most standardized Single-Sheet-Testers or Epstein Frames the characterization range is limited to 1.8 or 1.9 T, although saturation of most FeSi electrical steels exceeds 2.0 T. The saturation behavior can be modeled with consideration of the chemical composition according to [67] combined with a Fröhlich-Kennelly extrapolation approach. Thus, in the case of saturation, the model becomes an extreme of an ellipse, namely a circle. By an extrapolation of the three magnetization curves used to parameterize the model and the elliptical approach, the magnetization behavior up to full saturation can be described in the entire sheet plane.

**Figure 19:**
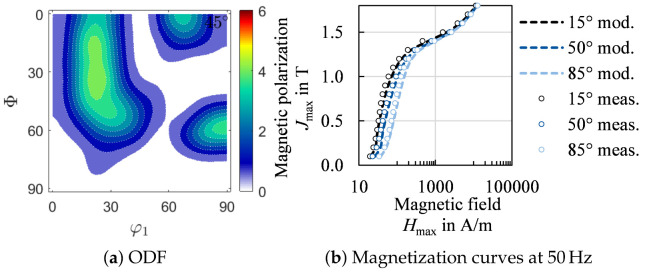
(**a**) Section $\varphi_{2}=45$° of measured ODF, (**b**) modeled and measured magnetization curves at 50 Hz in three directions of the M235-35A steel.

In Figure 19b, three exemplary magnetization curves are displayed, which are modeled with Equation (30). The modeled as well as the measured behavior are displayed. The proposed model can portray crossings of magnetization curves and can easily be implemented in finite-element magnetic field and flux simulations. In [23,68], it is described how magnetization curves can be reformulated to describe the reluctivity. With this, electromagnetic problems can be solved by means of the vector potential. In [68], the reluctivity surfaces for anisotropic magnetization behavior, as obtained by the presented model, are implemented. A modeled ODF or experimentally obtained ODF can both be used to parameterize the model and describe the anisotropic magnetization behavior.

### 7.2. Semi-Physical Loss Model

For the loss modeling of electrical steel, various approaches have been established over the years [69]. These models range from solely empirical models to physical models. The IEM model is an evolution of the Steinmetz and Bertotti model [70,71], where the total loss is separated into different loss components, which account for different loss mechanisms. The distinction of static and dynamic loss components and accounting for local and global eddy current effects as well as considering saturation effects allows a physical interpretation of calculated results for electrical machines.

The semi-physical IEM model is parameterized solely by magnetic measurements at various frequencies. However, the separate loss terms have, as explained in [70,72], a physical explanation and can thus, directly be linked to material parameters. Grain size, silicon content and sheet thickness are the most relevant parameters which affect the different loss components [3]. In comparison to Bertotti’s model, a term to account for nonlinear material behavior has been included in the IEM model to improve the modeling at high frequencies and high polarizations [71]. This is necessary, if the model is for example used in finite-element simulations of traction drives, because high power density requires high speed and consequently high magnetization frequencies. Since traction drives are speed variable, the entire frequency range up to the maximum speed is relevant to consider all operating points. The model can be described by the following equation,

(32) $$\begin{array}{c} P_{\mathrm{IEM}}=a_{1}B_{\max}^{\alpha +B_{\max}\beta }f+a_{2}B_{\max}^{2}f^{2}\left(1+a_{3}B_{\max}^{a_{4}}\right)+a_{5}{\left(B_{\max}f\right)}^{1.5} \end{array}$$

where $a_{1}$ to $a_{5}$, α and β can be identified by a parameter-identification procedure [72]. The classical eddy current loss depends on sheet thickness $d_{\mathrm{sheet}}$ and electric resistivity $\rho_{\mathrm{el}}$ which is directly linked to the chemical composition

(33) $$a_{2}=\frac{{\left(\pi d_{\mathrm{sheet}}\right)}^{2}}{6\rho_{\mathrm{el}}}\;$$

**Figure 20:**
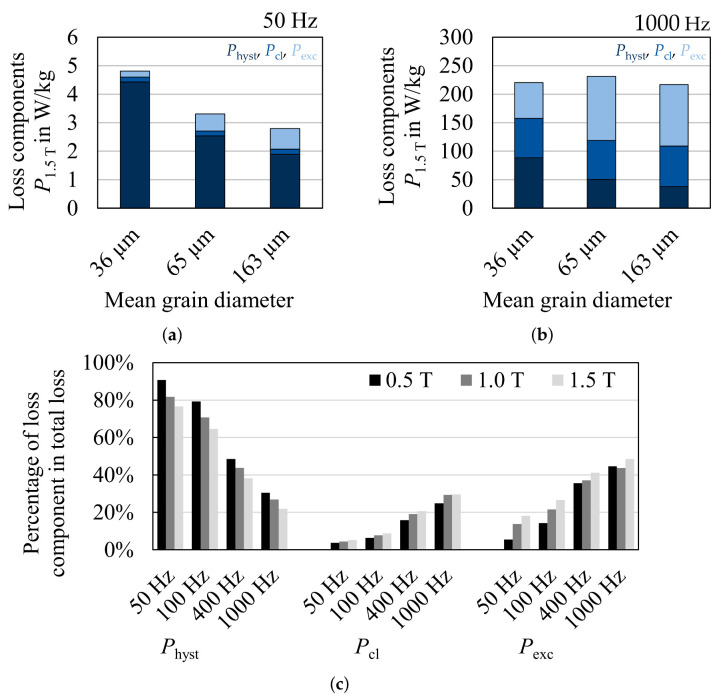
Loss component distribution at 1.5 T for three 0.25 mm thick non-oriented electrical steel grades at (**a**) 50 Hz and (**b**) 1000 Hz, and (**c**) loss component distribution for the sample with a mean grain size of 65 μm and thickness of 0.25 mm.

Thus, the alloying with silicon to increase the electrical resistivity and the reduction of sheet thickness are easy measures to reduce the high frequency loss in electrical steels. The hysteresis as well as the excess loss depend on the grain size [3,70]. With increasing grain size, the domain wall movement during the magnetization process is improved, as domain walls are less often pinned by defects such as grain boundaries. Moreover, the coercivity is decreased and the static loss component is reduced. The excess loss component increases with increasing grain size. Due to the less impeded domain wall movement during the magnetization process, the change in magnetic flux induces local eddy currents which cause the so-called excess losses. In Figure 20, a loss separation for three 0.25 mm iron silicon steels with the same chemical composition but different grain sizes are displayed at low, medium and high polarization levels and 50 Hz and 1000 Hz. The dominant impact of grain size is evident. At low frequencies, the coarse microstructure has fewer overall losses, but at increasing frequencies the loss component distribution changes and finer grained materials have almost identical overall losses. In Figure 20c, the relative shares of the loss components are displayed for the three materials. With increasing frequency and higher polarization the hysteresis loss decreases, while the eddy current loss increases and the excess loss slightly increases. This distribution depends on material parameters because the share of eddy current losses would be increased drastically with a higher sheet thickness or less silicon content. Apart from the material parameters discussed, the sheet thickness and residual stress state affects hysteresis and excess loss as well, even though the effect is by far less dominant than the presented relations.

The needed material parameters can either be experimentally characterized on samples which are magnetically characterized, or they can be transferred from the preceding models. In the second case, however, the parameter space for a certain alloy must be identified beforehand, especially regarding the nonlinear loss that need to be determined mathematically during the parameterization from magnetic measurements. The microstructural parameters can then be used to span a parameter space to characterize an alloy and estimate loss of grades with different grain size or thicknesses and therefore enable a tailor-made approach as presented by [73].

## 8. Conclusions

Material models and simulation tools are essential for process inventions and to enable tailor-made material development. We introduced and examined model approaches for the process chain of hot rolling, cold rolling, annealing, and blanking for high silicon non-oriented electrical steel. Moreover, models for the prediction of magnetization anisotropy as a function of texture and silicon content, as well as the prediction of magnetic loss affected by microstructure, thickness, and alloying content, were presented.

All described modeling approaches can consider heterogeneous microstructures that are typical for non-oriented electrical steel. As for all material models, the outcome strongly relies on how far the physical phenomena in each process step are understood as well as on the availability and quality of material parameters. Therefore, each material model can be improved by new or more detailed material characterization approaches.

For hot rolling, a concept of predicting grain size and its distribution for three positions in the hot strip, namely surface, intermediate and mid-layer is presented. For this, a good agreement with experimental data exists. Further improvements seem possible by refining the local strain and strain rate calculation in the layer model. Furthermore, changes in flow stress as a result of recovery, recrystallization, and grain growth could be considered by an individually adjusted flow stress model.

For the simulation of the microstructure evolution during cold rolling, the grain size distribution, either from simulation or from grain size measurements, and the measured hot strip texture are used. Moreover, selected textures can be used as initial input data to estimate their effects on the cold rolling texture. The combination of a macro model and a micro model with CPFEM predicted the cold rolling texture well.

For the annealing model, besides material parameters, the simulated or experimentally determined cold rolling microstructure (grain size, grain shape, dislocation density) and texture are the most important input parameters. Currently, the nucleation process during deformation and recovery is largely unknown in the literature and must therefore be approximated via experiments. Subsequently, the movement of grain boundaries is simulated based on different driving forces (stored elastic energy, curvature) as well as grain boundary characteristics (grain boundary misorientation angle, grain boundary energy, grain boundary mobility). Moreover, the model is written in such a way that additional findings regarding the grain boundary mobility distribution, triple junction drag, or nucleation can be implemented. The fit to experiments is already very good; however, some phenomena (nucleation, triple junction drag, grain boundary mobility distribution) are still approximated and leave room for future improvement of these simulations and underlying physics-based models.

In blanking, the homogeneous material model allows for the qualitative comparison of residual stress for different shear cutting parameters [22]. The comparison with the surface model used here shows, however, that quantitative values are strongly dependent on the applied material model. Validation of the surface model with experimental residual stress measurements should be pursued further to prove or falsify its benefit for the evaluation of residual stress by FEA.

Together, we believe the models presented here, covering the entire process chain, will enable a tailor-made material and process design and closed simulation chain.

## Figures and Tables

**Table 1:** Chemical composition of the investigated non-oriented electrical steel in the case studies.

Chemical Element	Si	Al	Mn	P	S	C	Fe
wt.%	3.16	0.89	0.17	0.07	0.003	0.002	balance

**Table 2:** Material data used in the microstructure model for hot rolling in Equations (7)–(10).

$c_{1}$	$c_{2}$	$c_{3}$	$c_{4}$	$c_{5}$	$c_{7}$	$c_{8}$	$c_{9}$
0.025	0.025	1.5	0.08	0.08	0.1	1	1
α	***M***	***A***	$d_{\mathrm{sp}}$	η	$\gamma_{\mathrm{GB}}$	$\gamma_{n}$	
0.5	3	0.5	2*b*	0.1	0.5	0.5	

**Table 3:** CPFEM phenopower law model parameters for the sample material a Fe3.2wt.%Si with a homogeneous and a banded microstructure.

Elastic parameters						
		C${}_{11}$	C${}_{12}$	C${}_{44}$		
		232.2 GPa	135.6 GPa	117.0 GPa		
**Plastic Parameters for Homogeneous Hot Strip Microstructure**						
slip system	${\dot{\gamma }}_{0}$	**s**	$\tau_{0}^{c}$	$\tau_{\infty }$	$\sigma_{h}$	**a**
{110}〈111〉	0.1 s^−1^	117	174.86 GPa	419.97 GPa	2.87 × 10^9^	1.60
{211}〈111〉	0.1 s^−1^	117	167.20 GPa	419.97 GPa	2.87 × 10^9^	1.60
**Plastic Parameters for Hot Strip with Banded Microstructure**						
slip system	${\dot{\gamma }}_{0}$	**s**	$\tau_{0}^{c}$	$\tau_{\infty }$	$\sigma_{h}$	**a**
{110}〈111〉	0.1 s^−1^	77	145.06 GPa	314.61 GPa	2.77 × 10^9^	2.12
{211}〈111〉	0.1 s^−1^	77	140.30 GPa	323.17 GPa	2.77 × 10^9^	2.12

**Table 4:** Input parameters and their origin for the microstructure generator for the annealing temperatures 900 °C, 1000 °C and 1100 °C.

Parameter	900 °C	1000 °C	1100 °C	Comment, Reference
NucleiDensity	0.01	0.0005	0.0004	nucleation vs. growth
NucleiSEE	1 × 10^10^ m^−2^	1 × 10^10^ m^−2^	1 × 10^10^ m^−2^	[14] and estimated
QualitativeSEE	3.75 x (Goss); x (γ-fiber); 5/8 x (α-fiber)			experiment
QuantitativeSEE	6.4 × 10^13^ m^−2^	6.4 × 10^13^ m^−2^	6.4 × 10^13^ m^−2^	[14] and estimated
SubGrainScatter	3°	3°	3°	estimated
Layer	Mid (texture)	Mid (texture)	Mid (texture)	measured
GrainShape	0.04	0.04	0.04	model maximum

**Table 5:** Input parameters and their origin for the GraGLeS2D+ level-set model for the annealing temperatures 900 °C, 1000 °C, and 1100 °C.

Parameter	900 °C	1000 °C	1100 °C	Comment, Reference
HAGB_Energy	1.2 Js/m^2^	1.2 Js/m^2^	1.2 Js/m^2^	[52]
HAGB_Mobility	0.99 × 10^−11^ m^4^/Js	3.06 × 10^−11^ m^4^/Js	8.02 × 10^−11^ m^4^/Js	[53,54] and experiment
DislocEnPerM	$\frac{1}{2}Gb^{2}$; G = 83 GPa; $b_{111}=\frac{a}{2}\sqrt{h^{2}+k^{2}+l^{2}}$; a = 2.86 *Å*			Thermo-Calc
TripleJunctionDrag	100	100	100	estimated
NrOfPPG	15	15	15	[55]

**Table 6:** Material properties of the examined non-oriented electrical steel Fe3.2wt.%Si.

Uniform Expansion	Tensile Strength	Yield Strength
13.62 %	511.61 MPa	391.65 MPa
**E-Modulus**	**Hardening Exponent**	**Normal Anisotropy**
147.83 MPa	0.10	1.16

## Data Availability

The datasets generated and analyzed during the current study are available from the corresponding author on reasonable request.

## Symbols

**Symbol**	**Definition**
*a*	material parameter representing rate sensitivity of slip system
$a_{1}$ to $a_{5}$	material parameter for magnetic loss model
*A*	activation area
$A_{f}$	material parameter for Freiberg flow stress law
$A_{\theta }$	magnetic A-Parameter
α	dislocation to dislocation interaction constant
$\alpha_{j}$	angles of the respective layer
α,β	slip system or material parameter for magnetic loss model
*b*	Burgers vector
bcc	body centered cubic
$B_{\max}$	maximal magnetic flux density
$c_{1}$	grain size effect parameter
$c_{2}$	dislocation density effect parameter
$c_{3}$	spontaneous annihilation effect parameter
$c_{4}$	annihilation by diffusion effect parameter
$c_{5}$	speed of dislocations effect parameter
$c_{6}$	mobile dislocations effect parameter
$c_{7}/c_{8}$	shearing effect parameters
$c_{9}$	activation volume effect parameter
CPFEM	crystal plasticity finite-element method
*d*	grain size
$d_{\mathrm{sheet}}$	sheet thickness
$d_{sp}$	critical distance for spontaneous annihilation
$D_{s}$	self-diffusion coefficient
η	scale factor
*f*	frequency
$f_{n}$	nucleation frequency
*F*	deformation gradient
*G*	shear modulus
γ	grain boundary energy
$\gamma_{GB}$	surface energy of the new grain boundary
$\gamma_{n}$	surface energy of the nuclei
${\dot{\gamma }}^{\alpha }$	shear rate
${\dot{\gamma }}_{{}_{0}}$	reference shear rate
*h*	height of strip
$h_{P}$	Planck’s quantum of action
$h_{{}_{tot}}$	total height of the strip
$h_{1}$	smallest roll gap height
*H*	displacement gradient
$H_{\mathrm{texture}}$	texture related magnetic field strength
HAGB	high-angle grain boundary
i	counting index
j	index indicating individual layer
*J*	magnetic polarization
*k*	Boltzmann constant
κ	yield across grain boundaries
$l_{d}$	projected arc contact between rolls and metal
$L_{p}$	plastic velocity gradient
*M*	Taylor factor
*m*	unit vector for the slip direction
$m_{i}$	material parameters for Freiberg flow stress law
$\mu_{GB}$	high-angle grain boundary mobility
*n*	normal to the slip plan of slip system α
$n_{r}$	rotational frequency
*N*	total number of grains
$\dot{N}$	nucleation rate
$N_{\mathrm{ig}}$	number of inner grains
$N_{\mathrm{sg}}$	number of surface grains
ND	normal direction
NO	non-oriented
ω	weighting factor
Ω	computational domain
ODF	orientation distribution function
$P_{{}_{RX}}$	pressure on an individual grain boundary
φ	logarithmic strain
Φ(t,x)	level-set function
$\dot{\varphi }$	logarithmic rate
${\dot{\varphi }}_{{}_{mean}}$	mean logarithmic rate
$q_{{}_{\alpha \beta }}$	measure for latent hardening
$Q_{{}_{diff}}$	self-diffusion activation energy
*R*	universal gas constant
$R_{N}$	nucleation radius
$R_{r}$	roll radius
RD	rolling direction
RVE	representative volume element
ρ	density
$\rho_{{}_{el}}$	electric resistivity
*S*	interface
SEE	dislocation density
$\sigma_{f}$	flow stress
$\sigma_{f}\;\mathrm{mean}$	mean flow stress
$\sigma_{h}$	initial hardening stress
$\sigma_{\mathrm{ig}}$	flow stress of inner grains
$\sigma_{\mathrm{sg}}$	flow stress of surface grains
$\tau^{\alpha }$	resolved shear stress of slip system α
$\tau_{c}^{\alpha }$	critical resolved shear stress of slip system α
$\tau_{s}$	saturated flow stress
$\tau_{R}$	resolved shear stress
TD	transversal direction
θ	misorientation angle or angle relative to rolling direction
ϑ	temperature
$u_{a},u_{b}$	history of the displacement vectors
*v*	velocity
$v_{f}$	Debye-frequency
*w*	slip hardening exponent
wt.%	weight percent
*x*	position coordinate in the roll gap
$X_{a},X_{b}$	position vectors of the opposite node pairs of the RVE
XRD	X-ray diffraction
